# Supplementary material for: Discovery and application of insertion-deletion (INDEL) polymorphisms for QTL mapping of early life-history traits in Atlantic salmon
Source: BMC Genomics. 2010 Mar 8;11:156. doi: 10.1186/1471-2164-11-156 (PMC2838853; doi:10.1186/1471-2164-11-156)
Supplement: Additional file 2 — Information on developed 76 locus single-run INDEL panel in Atlantic salmon. Information on fluorescence labeling, primer concentrations, PCR pooling and links to alignments, INDEL motifs and GENESCAN (Burge and Karlin 1997) predictions of genes/exons are available in html format. [file 1471-2164-11-156-S2.ZIP › Additionalfile2/table20196.html]

Contig Alignment

```
Base	A B C D E   
1	G . . . .    
2	G . . . .    
3	G . . . .    
4	G . . . .    
5	C . . . .    
6	A . . . .    
7	T . . . .    
8	G . . . .    
9	G . . . .    
10	G . . . .    
11	T . . . .    
12	A . . . .    
13	T . . . .    
14	A . . . .    
15	T . . . .    
16	G . . . .    
17	A . . . .    
18	C . . . .    
19	C . . . .    
20	C . . . .    
21	A . . . .    
22	G . . . .    
23	G . . . .    
24	A . . . .    
25	G . . . .    
26	A . . . .    
27	A . . . .    
28	C . . . .    
29	C . . . .    
30	T . . . .    
31	G . . . .    
32	A . . . .    
33	A . . . .    
34	T . . . .    
35	T . . . .    
36	T . . . .    
37	A . . . .    
38	A . . . .    
39	A . . . .    
40	T . . . .    
41	T . . . .    
42	C . . . .    
43	A . . . .    
44	A . . . .    
45	T . . . .    
46	A . . . .    
47	G . . . .    
48	A . . . .    
49	T . . . .    
50	A . . . .    
51	G . . . .    
52	T . . . .    
53	T . . . .    
54	G . . . .    
55	A . . . .    
56	A . . . .    
57	T . . . .    
58	G . . . .    
59	C . . . .    
60	A . . . .    
61	T . . . .    
62	G . . . .    
63	T . . . .    
64	G . . . .    
65	C . . . .    
66	C . . . .    
67	T . . . .    
68	C . . . .    
69	A . . . .    
70	A . . . .    
71	T . . . .    
72	A . . . .    
73	A . . . .    
74	A . . . .    
75	A . . . .    
76	A . . . .    
77	A . . . .    
78	A . . . .    
79	C . . . .    
80	A . . . .    
81	A . . . .    
82	A . . . .    
83	T . . . .    
84	T . . . .    
85	C . . . .    
86	C . . . .    
87	A . . . .    
88	A . . . .    
89	C . . . .    
90	T . . . .    
91	T . . . .    
92	C . . . .    
93	A . . . .    
94	G . . . .    
95	A . . . .    
96	T . . . .    
97	G . . . .    
98	C . . . .    
99	G . . . .    
100	T . . . .    
101	G . . . .    
102	C . . . .    
103	A . . . .    
104	T . . . .    
105	A . . . .    
106	G . . . .    
107	C . . . .    
108	T . . . .    
109	G . . . .    
110	A . . . .    
111	T . . . .    
112	G . . . .    
113	T . . . .    
114	T . . . .    
115	G . . . .    
116	A . . . .    
117	T . . . .    
118	T . . . .    
119	T . . . .    
120	A . . . .    
121	A . . . .    
122	A . . . .    
123	G . . . .    
124	A . . . .    
125	A . . . .    
126	A . . . .    
127	A . . . .    
128	C . . . .    
129	G . . . .    
130	T . . . .    
131	C . . . .    
132	A . . . .    
133	A . . . .    
134	G . . . .    
135	T . . . .    
136	T . . . .    
137	T . . . .    
138	C . . . .    
139	A . . . .    
140	T . . . .    
141	G . . . .    
142	C . . . .    
143	A . . . .    
144	C . . . .    
145	A . . . .    
146	T . . . .    
147	A . . . .    
148	T . . . .    
149	T . . . .    
150	T . . . .    
151	G . . . .    
152	T . . . .    
153	A . . . .    
154	A . . . .    
155	T . . . .    
156	T . . . .    
157	T . . . .    
158	G . . . .    
159	T . . . .    
160	T . . . .    
161	T . . . .    
162	T . . . .    
163	T . . . .    
164	G . . . .    
165	A . . . .    
166	A . . . .    
167	T . . . .    
168	T . . . .    
169	A . . . .    
170	T . . . .    
171	G . . . .    
172	T . . . .    
173	A . . . .    
174	A . . . .    
175	T . . . .    
176	T . . . .    
177	T . . . .    
178	T . . . .    
179	A . . . .    
180	G . . . .    
181	A . . . .    
182	G . . . .    
183	A . . . .    
184	T . . . .    
185	A . . . .    
186	T . . . .    
187	T . . . .    
188	A . . . .    
189	C . . . .    
190	A . . . .    
191	A . . . .    
192	T . . . .    
193	G . . . .    
194	A . . . .    
195	G . . . .    
196	A . . . .    
197	G . . . .    
198	G . . . .    
199	T . . . .    
200	T . . . .    
201	T . . . .    
202	G . . . .    
203	G . . . .    
204	T . . . .    
205	T . . . .    
206	A . . . .    
207	A . . . .    
208	A . . . .    
209	A . . . .    
210	G . . . .    
211	A . . . .    
212	T . . . .    
213	G . . . .    
214	T . . . .    
215	T . . . .    
216	T . . . .    
217	G . . . .    
218	A . . . .    
219	A . . . .    
220	A . . . .    
221	A . . . .    
222	A . . . .    
223	T . . . .    
224	G . . . .    
225	T . . . .    
226	A . . . .    
227	A . . . .    
228	G . . . .    
229	T . . . .    
230	G . . . .    
231	A . . . .    
232	A . . . .    
233	A . . . .    
234	C . . . .    
235	C . . . .    
236	T . . . .    
237	T . . . .    
238	G . . . .    
239	T . . . .    
240	G . . . .    
241	T . . . .    
242	T . . . .    
243	G . . . .    
244	A . . . .    
245	A . . . .    
246	C . . . .    
247	C . . . .    
248	A . . . .    
249	T . . . .    
250	G . . . .    
251	G . . . .    
252	T . . . .    
253	G . . . .    
254	C . . . .    
255	A . . . .    
256	C . . . .    
257	T . . . .    
258	T . . . .    
259	C . . . .    
260	G . . . .    
261	T . . . .    
262	T . . . .    
263	C . . . .    
264	A . . . .    
265	T . . . .    
266	T . . . .    
267	G . . . .    
268	T . . . .    
269	T . . . .    
270	A . . . .    
271	T . . . .    
272	T . . . .    
273	C . . . .    
274	A . . . .    
275	C . . . .    
276	T . . . .    
277	G . . . .    
278	T . . . .    
279	A . . . .    
280	C . . . .    
281	A . . . .    
282	G . . . .    
283	T . . . .    
284	T . . . .    
285	A . . . .    
286	C . . . .    
287	T . . . .    
288	T . . . .    
289	C . . . .    
290	T . . . .    
291	C . . . .    
292	T . . . .    
293	A . . . .    
294	T . . . .    
295	T . T T .    
296	T . T T .    
297	T . T T .    
298	T . T T .    
299	G . G G .    
300	T . T T .    
301	T . T T .    
302	C . C C .    
303	A . A A .    
304	T . T T .    
305	A . A A .    
306	A . A A .    
307	C . C C .    
308	T . T T .    
309	C . C C .    
310	A . A A .    
311	T . T T .    
312	C . C C .    
313	G . G G .    
314	A . T T .    
315	C C C C .    
316	A A A A .    
317	A A A A .    
318	C C C C .    
319	A A A A .    
320	T T T T .    
321	T T T T .    
322	C C C C .    
323	C C C C .    
324	A A A A .    
325	T T T T .    
326	A A A A .    
327	G G G G .    
328	A A A A .    
329	G G G G .    
330	C C C C .    
331	A A A A .    
332	G G G G .    
333	C C C C .    
334	T T T T .    
335	T T T T .    
336	G G G G .    
337	T T T T .    
338	T T T T .    
339	C C C C .    
340	T T T T .    
341	T T T T .    
342	A A A A .    
343	T T T T .    
344	T T T T .    
345	T T T T .    
346	T T T T .    
347	T T T T .    
348	T T T T .    
349	T T T T .    
350	A A A A .    
351	A A A A .    
352	C C C C .    
353	A A A A .    
354	T T T T .    
355	T T T T .    
356	C C C C .    
357	A A A A .    
358	A A A A .    
359	A A A A .    
360	C C C C .    
361	T T T T .    
362	T T T T .    
363	T T T T .    
364	T T T T .    
365	G G G G .    
366	G G G G .    
367	T T T T .    
368	A A A A .    
369	T T T T .    
370	A A A A .    
371	C C C C .    
372	G G G G .    
373	T T T T .    
374	A A A A .    
375	A A A A .    
376	G G G G .    
377	G G G G .    
378	G G G G .    
379	G G G G .    
380	C C C C .    
381	A A A A .    
382	A A A A .    
383	T T T T .    
384	C C C C .    
385	A A A A .    
386	T T T T .    
387	T T T T .    
388	T T T T .    
389	T T T T .    
390	G G G G .    
391	T T T T .    
392	A A A A .    
393	T T T T .    
394	A A A A .    
395	T T T T .    
396	A A A A .    
397	T T T T .    
398	A A A A .    
399	G G G G .    
400	T T T T .    
401	T T T T .    
402	T T T T .    
403	A A A A .    
404	G G G G .    
405	G G G G .    
406	C C C C .    
407	A A A A .    
408	G G G G .    
409	T T T T .    
410	T T T T .    
411	A A A A .    
412	T T T T .    
413	A A A A .    
414	T T T T .    
415	C C C C .    
416	C C C C .    
417	T T T T .    
418	T T T T .    
419	A A A A .    
420	C C C C .    
421	A A A A .    
422	A A A A .    
423	G G G G .    
424	G G G G .    
425	A A A A .    
426	A A A A .    
427	G G G G .    
428	A A A A .    
429	T T T T .    
430	G G G G .    
431	C C C C .    
432	A A A A .    
433	T T T T .    
434	T T T T .    
435	T T T T .    
436	G G G G .    
437	T T T T .    
438	T T T T .    
439	G G G G .    
440	T T T T .    
441	G G G G .    
442	C C C C .    
443	T T T T .    
444	A A A A .    
445	C C C C .    
446	A A A A A    
447	G G G G G    
448	A A A A A    
449	A A A A A    
450	A A A A A    
451	T T T T T    
452	T T T T T    
453	T T T T T    
454	G G G G G    
455	C C C C C    
456	A A A A A    
457	T T T T T    
458	C C C C C    
459	A A A A A    
460	G G G G G    
461	T T T T T    
462	G G G G G    
463	T T T T T    
464	G G G G G    
465	C C C C C    
466	A A A A A    
467	A A A A A    
468	A A A A A    
469	C C C C C    
470	T T T T T    
471	A A A A A    
472	G G G G G    
473	C C C C C    
474	C C C C C    
475	T T T T T    
476	C C C C C    
477	T T T T T    
478	C C C C C    
479	A A A A A    
480	G G G G G    
481	G G G G G    
482	T T T T T    
483	C C C C C    
484	A A A A A    
485	G G G G G    
486	A A A A A    
487	T T T T T    
488	A A A A A    
489	T T T T T    
490	T T T T T    
491	C C C C C    
492	T T T T T    
493	C C C C C    
494	A A A A A    
495	G G G G G    
496	G G G G G    
497	T T T T T    
498	T T T T T    
499	C C C C C    
500	G G G G G    
501	T T T T T    
502	A A A A A    
503	T T T T T    
504	A A A A A    
505	C C C C C    
506	A A A A A    
507	A A A A A    
508	G G G G G    
509	G G G G G    
510	A A A A A    
511	C C C C C    
512	A A A A A    
513	T T T T T    
514	T T T T T    
515	A A A A A    
516	A A A A A    
517	G G G G G    
518	A A A A A    
519	A A A A A    
520	A A A A A    
521	T T T T T    
522	T T T T T    
523	A A A A A    
524	A A A A A    
525	T T T T T    
526	C C C C C    
527	A A A A A    
528	A A A A A    
529	A A A A A    
530	A A A A A    
531	T T T T T    
532	A A A A A    
533	A A A A A    
534	C C C C C    
535	C C C C C    
536	A A A A A    
537	A A A A A    
538	T T T T T    
539	T T T T T    
540	T T T T T    
541	G G G G G    
542	A A A A A    
543	T T T T T    
544	G G G G G    
545	A A A A A    
546	C C C C C    
547	C C C C C    
548	A A A A A    
549	A A A A A    
550	T T T T T    
551	A A A A A    
552	A A A A A    
553	G G G G G    
554	C C C C C    
555	A A A A A    
556	C C C C C    
557	A A A A A    
558	A A A A A    
559	T T T T T    
560	T T T T T    
561	T T T T T    
562	A A A A A    
563	G G G G G    
564	T T T T T    
565	T T T T T    
566	A A A A A    
567	T T T T T    
568	C C C C C    
569	T T T T T    
570	G G G G G    
571	A A A A A    
572	A A A A A    
573	A A A A A    
574	A A A A A    
575	T T T T T    
576	A A A A A    
577	T T T T T    
578	T T T T T    
579	A A A A A    
580	C C C C C    
581	T T T T T    
582	T T T T T    
583	G G G G G    
584	T T T T T    
585	T T T T T    
586	A A A A A    
587	C C C C C    
588	A A A A A    
589	A A A A A    
590	A A A A A    
591	C C C C C    
592	A A A A A    
593	G G G G G    
594	A A A A A    
595	A A A A A    
596	A A A A A    
597	G G G G G    
598	G G G G G    
599	G G G G G    
600	A A A A A    
601	A A A A A    
602	T T T T T    
603	G G G G G    
604	T T T T T    
605	A A A A A    
606	T T T T T    
607	T T T T T    
608	T T T T T    
609	A A A A A    
610	A A A A A    
611	T T T T T    
612	T T T T T    
613	G G G G G    
614	C C C C C    
615	C C C C C    
616	T T T T T    
617	T T T T T    
618	A A A A A    
619	T T T T T    
620	A A A A A    
621	C C C C C    
622	T T T T T    
623	G G G G G    
624	C C C C C    
625	C C C C C    
626	T T T T T    
627	A A A A A    
628	C C C C C    
629	T T T T T    
630	T T T T T    
631	A A A A A    
632	T T T T T    
633	T T T T T    
634	T T T T T    
635	G G G G G    
636	T T T T T    
637	T T T T T    
638	T T T T T    
639	T T T T T    
640	T T T T T    
641	C C C C C    
642	T T T T T    
643	G G G G G    
644	C C C C C    
645	T T T T T    
646	T T T T T    
647	T T T T T    
648	A A A A A    
649	T T T T T    
650	A A A A A    
651	A A A A A    
652	T T T T T    
653	T T T T T    
654	G G G G G    
655	T T T T T    
656	A A A A A    
657	A A A A A    
658	T T T T T    
659	A A A A A    
660	A A A A A    
661	T T T T T    
662	G G G G G    
663	A A A A A    
664	T T T T T    
665	C C C C C    
666	T T T T T    
667	G G G G G    
668	T T T T T    
669	C C C C C    
670	A A A A A    
671	A A A A A    
672	C T T T T    
673	. T T T T    
674	. T T T T    
675	. A A A A    
676	. C C C C    
677	. T T T T    
678	. T T T T    
679	. G G G G    
680	. T T T T    
681	. T T T T    
682	. T T T T    
683	. T T T T    
684	. T T T T    
685	. T A T A  2	 transversion  
686	. A A A A    
687	. A A A A    
688	. A A A A    
689	. A A A A    
690	. A A A A    
691	. A A A A    
692	. A A A A    
693	. G G G G    
694	. A A A A    
695	. A A A A    
696	. T T T T    
697	. T T T T    
698	. A A A A    
699	. C C C C    
700	. A A A A    
701	. A A A A    
702	. T T T T    
703	. T T T T    
704	. A A A A    
705	. T T T T    
706	. T T T T    
707	. T T T T    
708	. A A A A    
709	. A A A A    
710	. T T T T    
711	. T T T T    
712	. A A A A    
713	. C C C C    
714	. T T T T    
715	. G G G G    
716	. T T T T    
717	. A A A A    
718	. A A A A    
719	. C C C C    
720	. T T T T    
721	. C C C C    
722	. C C C C    
723	. C C C C    
724	. C C C C    
725	. A A A A    
726	. A A A A    
727	. T T T T    
728	. C C C C    
729	. C C C C    
730	. T T T T    
731	. T T T T    
732	. T T T T    
733	. G G G G    
734	. A A A A    
735	. C C C C    
736	. A A A A    
737	. T T T T    
738	. A A A A    
739	. T T T T    
740	. C C C C    
741	. A A A A    
742	. C C C C    
743	. T T T T    
744	. C C C C    
745	. A A A A    
746	. A A A A    
747	. T T T T    
748	. C C C C    
749	. T T T T    
750	. A A A A    
751	. A A A A    
752	. A A A A    
753	. C C C C    
754	. C C C C    
755	. T T T T    
756	. C C C C    
757	. T T T T    
758	. G G G G    
759	. T T T T    
760	. T T T T    
761	. G G G G    
762	. A A A A    
763	. T T T T    
764	. C C C T    
765	. T T T T    
766	. G G G G    
767	. A A A A    
768	. T T T T    
769	. T T T T    
770	. T T T T    
771	. A A A A    
772	. T T T T    
773	. A A A A    
774	. T T T T    
775	. T T T T    
776	. T T T T    
777	. A A A A    
778	. T T T T    
779	. T T T T    
780	. T T T T    
781	. G G G G    
782	. A A A A    
783	. G G G G    
784	. A A A A    
785	. G G G G    
786	. T T T T    
787	. T T T T    
788	. C C C C    
789	. A A A A    
790	. C C C C    
791	. A A A A    
792	. T T T T    
793	. T T T T    
794	. G G G G    
795	. T T T T    
796	. T T T T    
797	. A A A A    
798	. C C C C    
799	. T T T T    
800	. T T T T    
801	. A A A A    
802	. C C C C    
803	. A A A A    
804	. A A A A    
805	. C C C C    
806	. C C C C    
807	. A A A A    
808	. C C C C    
809	. T T T T    
810	. G G G G    
811	. T T T T    
812	. T T T T    
813	. T T T T    
814	. C C C C    
815	. T T T T    
816	. G G G G    
817	. A A A A    
818	. G G G G    
819	. T T T T    
820	. A A A A    
821	. A A A A    
822	. T T T T    
823	. G G G G    
824	. C C C C    
825	. A A A A    
826	. G G G G    
827	. T T T T    
828	. C C C C    
829	. T T T T    
830	. - A - A  2	 indel  
831	. - T - T  2	 indel  
832	. G G G G    
833	. T T T T    
834	. T T T T    
835	. T T T T    
836	. T T T T    
837	. T T T T    
838	. A A A A    
839	. T T T T    
840	. C C C C    
841	. T T T T    
842	. G G G G    
843	. T T T T    
844	. T T T T    
845	. A A A A    
846	. T T T T    
847	. A A A A    
848	. T T T T    
849	. T T T T    
850	. G G G G    
851	. A A A A    
852	. A A A A    
853	. A A A A    
854	. G G G G    
855	. G G G G    
856	. C C C C    
857	. A A A A    
858	. T T T T    
859	. T T T T    
860	. C C C C    
861	. A A A A    
862	. T T T T    
863	. T T T T    
864	. G G G G    
865	. A A A A    
866	. A A A A    
867	. G G G G    
868	. T T T T    
869	. A A A A    
870	. G G G G    
871	. A A A A    
872	. A A A A    
873	. A A A A    
874	. T T T T    
875	. C C C C    
876	. A A A A    
877	. C C C C    
878	. T T T T    
879	. G G G G    
880	. G G G G    
881	. A A A A    
882	. A A A A    
883	. A A A A    
884	. G G G G    
885	. C C C C    
886	. A A A A    
887	. T T T T    
888	. T T T T    
889	. T T T T    
890	. C C C C    
891	. A A A A    
892	. G G G G    
893	. C C C C    
894	. C C C C    
895	. C C C C    
896	. T T T T    
897	. G G G G    
898	. T T T T    
899	. A A A A    
900	. A A A A    
901	. T T T T    
902	. A A A A    
903	. T T T T    
904	. G G G G    
905	. A A A A    
906	. A A A A    
907	. T T T T    
908	. G G G G    
909	. A A A A    
910	. A A A A    
911	. G G G G    
912	. A A A A    
913	. A A A A    
914	. C C C C    
915	. T T T T    
916	. G G G G    
917	. G G G G    
918	. T T T T    
919	. G G G G    
920	. T T T T    
921	. G G G G    
922	. T T T T    
923	. . A A A    
924	. . C C C    
925	. . T T T    
926	. . C C C    
927	. . A A A    
928	. . A A A    
929	. . G G G    
930	. . T T T    
931	. . C C C    
932	. . T T T    
933	. . C C C    
934	. . C C C    
935	. . A A A    
936	. . C C C    
937	. . T T T    
938	. . T T T    
939	. . G G G    
940	. . T T T    
941	. . C C C    
942	. . C C C    
943	. . T T T    
944	. . T T T    
945	. . A A A    
946	. . A A A    
947	. . A A A    
948	. . T T T    
949	. . T T T    
950	. . T T T    
951	. . A A A    
952	. . G G G    
953	. . T T T    
954	. . A A A    
955	. . T T T    
956	. . T T T    
957	. . C C C    
958	. . A A A    
959	. . T T T    
960	. . T T T    
961	. . C C C    
962	. . C C C    
963	. . A A A    
964	. . A A A    
965	. . A G G    
966	. . . A A    
967	. . . A A    
968	. . . A A    
969	. . . A A    
970	. . . T T    
971	. . . G G    
972	. . . G G    
973	. . . T T    
974	. . . T T    
975	. . . T T    
976	. . . T T    
977	. . . C C    
978	. . . A A    
979	. . . A A    
980	. . . A A    
981	. . . T T    
982	. . . G G    
983	. . . T T    
984	. . . T T    
985	. . . G G    
986	. . . A A    
987	. . . T T    
988	. . . G G    
989	. . . C C    
990	. . . C C    
991	. . . T T    
992	. . . T T    
993	. . . A A    
994	. . . A A    
995	. . . A A    
996	. . . T T    
997	. . . T T    
998	. . . A A    
999	. . . T T    
1000	. . . G G    
1001	. . . C C    
1002	. . . A A    
1003	. . . A A    
1004	. . . T T    
1005	. . . G G    
1006	. . . A A    
1007	. . . A A    
1008	. . . A A    
1009	. . . G G    
1010	. . . T T    
1011	. . . C C    
1012	. . . A A    
1013	. . . T T    
1014	. . . G G    
1015	. . . C C    
1016	. . . A A    
1017	. . . T T    
1018	. . . G G    
1019	. . . T T    
1020	. . . G G    
1021	. . . T T    
1022	. . . T T    
1023	. . . A A    
1024	. . . A A    
1025	. . . T T    
1026	. . . T T    
1027	. . . A A    
1028	. . . T T    
1029	. . . C C    
1030	. . . A A    
1031	. . . G G    
1032	. . . T T    
1033	. . . G G    
1034	. . . T T    
1035	. . . T T    
1036	. . . T T    
1037	. . . G G    
1038	. . . A A    
1039	. . . T T    
1040	. . . T T    
1041	. . . T T    
1042	. . . G G    
1043	. . . T T    
1044	. . . G G    
1045	. . . T T    
1046	. . . A A    
1047	. . . T T    
1048	. . . T T    
1049	. . . T T    
1050	. . . G G    
1051	. . . T T    
1052	. . . T T    
1053	. . . G G    
1054	. . . T T    
1055	. . . G G    
1056	. . . T T    
1057	. . . G G    
1058	. . . C C    
1059	. . . C C    
1060	. . . T T    
1061	. . . A A    
1062	. . . T T    
1063	. . . T T    
1064	. . . A A    
1065	. . . A A    
1066	. . . A A    
1067	. . . A A    
1068	. . . C C    
1069	. . . C C    
1070	. . . A A    
1071	. . . G G    
1072	. . . T T    
1073	. . . T T    
1074	. . . T T    
1075	. . . A A    
1076	. . . A A    
1077	. . . G G    
1078	. . . C G    
1079	. . . . A    
1080	. . . . A    
1081	. . . . T    
1082	. . . . G    
1083	. . . . T    
1084	. . . . G
```
